# Photochromic switching of the DNA helicity induced by azobenzene derivatives

**DOI:** 10.1038/srep28605

**Published:** 2016-06-24

**Authors:** Marco Deiana, Ziemowit Pokladek, Joanna Olesiak-Banska, Piotr Młynarz, Marek Samoc, Katarzyna Matczyszyn

**Affiliations:** 1 Advanced Materials Engineering and Modelling Group, Faculty of Chemistry, Wroclaw University of Science and Technology, Wyb. Wyspianskiego 27, 50-370 Wroclaw, Poland; 2 Department of Bioorganic Chemistry, Faculty of Chemistry, Wroclaw University of Science and Technology, Wyb. Wyspianskiego 27, 50-370 Wroclaw, Poland

## Abstract

The photochromic properties of azobenzene, involving conformational changes occurring upon interaction with light, provide an excellent tool to establish new ways of selective regulation applied to biosystems. We report here on the binding of two water-soluble 4-(phenylazo)benzoic acid derivatives (Azo-2N and Azo-3N) with double stranded DNA and demonstrate that the photoisomerization of Azo-3N leads to changes in DNA structure. In particular, we show that stabilization and destabilization of the B-DNA secondary structure can be photochemically induced *in situ* by light. This photo-triggered process is fully reversible and could be an alternative pathway to control a broad range of biological processes. Moreover, we found that the bicationic Azo-3N exhibited a higher DNA-binding constant than the monocationic Azo-2N pointing out that the number of positive charges along the photosensitive polyamines chain plays a pivotal role in stabilizing the photochrome-DNA complex.

Control over DNA morphology is an important objective in various fundamental studies and applications, such as DNA-based nanomaterial devices and regulation of biological processes^1^,^2^,^3^,^4^,^5^,^6^. Several strategies to control *in vitro* DNA compaction rely either on the use of cationic molecules (e.g. polyamines, surfactants, nanoparticles, polymers and vesicles) able to neutralize DNA negative charges or on creating unfavorable contacts with the solvent (e.g. polyethylene glycol (PEG), precipitation in alcohol and solvents with low dielectric constants)^6^,^7^,^8^,^9^,^10^,^11^,^12^,^13^,^14^,^15^. Naturally occurring polyamines are abundant in living cells and play a pivotal role in maintaining cellular DNA in compact state thanks to multiply protonated amino groups^16^,^17^. As a result, natural or synthetic polyamines are frequently used as compacting agents in transfection applications^17^,^18^,^19^,^20^,^21^,^22^. However, once the DNA-polyamine complex is formed, its decomposition requires dramatic changes in the chemical composition of the samples^1^,^23^,^24^. An alternative strategy to trigger DNA compaction and decompaction, relies on the use of photoresponsive compacting agents, sensitive to light illumination, based on azobenzene moiety^6^,^17^,^25^,^26^. However, polyamines are not only responsible for inducing DNA condensation but also for promoting intrinsic changes in the DNA morphology^27^,^28^,^29^,^30^,^31^. Of increasing interest is the B-to-A-DNA transition and the biological role that A-DNA plays in cells^32^. A-like DNA is considered to be the outcome in many DNA-protein interactions or the result of the complexation with various biologically important ligands and counterions^32^,^33^,^34^,^35^,^36^ Moreover, RNA/DNA helices, for instance those necessary for transcription, exist in an A-like shape due to the incapability of the RNA/DNA helices to exist in a B-like form because of van der Waals crowding of the ribose C2-oxygen of the RNA^37^,^38^. It is also known that the B-to-A transition is responsible for protecting DNA from damaging agents such as heat and desiccation^39^. It turns out that modulating the intrinsic DNA secondary structure, by using non-invasive external stimuli, such as light, is a useful and needed strategy to trigger a broad range of bio-events.

In this contribution, we report the synthesis and characterization of photoswitchable DNA binding of two photochromic, water soluble 4-(phenylazo)benzoic acid derivatives based on azobenzene motif, denoted as Azo-2N and Azo-3N (Fig. 1), containing different linear unsubstituted polyamines moieties (see Supporting Information SI, p. S2 for details) and we show that both the Azo-3N’s isomers bind to the duplex with remarkably different binding affinity. Furthermore, we demonstrate that the intrinsic DNA chirality can be photochemically reversibly modulated by switching the shape of the non-covalently bound photochrome.

## Results and Discussion

**Figure 2:**
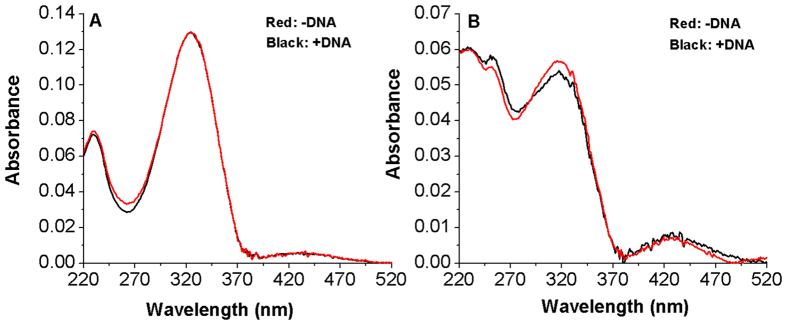
Absorption spectra of Azo-2N *trans* (**A**) and *cis* (**B**) in the absence (red line) and in the presence (black line) of *ds*-DNA. The concentrations of Azo-2N and DNA were 1 × 10^−5^ M in 10 mM sodium cacodylate trihydrate (pH 7.25).

The absorption spectra of the studied azobenzene derivatives in the *trans* form are mostly constituted of two bands: one of strong intensity located in the UV and the other, of weaker intensity, in the visible spectral region. The occurrence of such bands can be assigned to π-π^*^ and n-π* electronic transitions of the azobenzene moiety^40^. When DNA is added to Azo-2N, in both the *trans* and *cis* form, no appreciable interactions are observed, their spectra being virtually superimposable (Fig. 2). Since no interactions, within the experimental conditions used, were observed for Azo-2N-DNA in the UV-Vis spectra, no further experiments were carried out.

**Figure 3:**
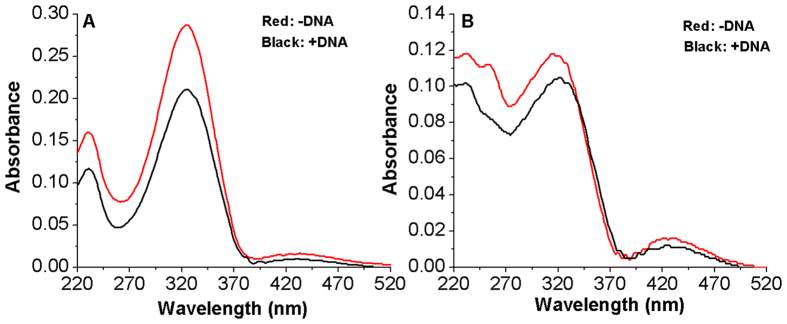
Absorption spectra of Azo-3N *trans* (**A**) and *cis* (**B**) in the absence (red line) and in the presence (black line) of *ds*-DNA. The concentration of Azo-3N and DNA was 2 × 10^−5^ M in 10 mM sodium cacodylate trihydrate (pH 7.25).

On the contrary, the addition of DNA to Azo-3N in both its isomeric forms promotes a strong hypochromic effect, clearly pointing out the occurrence of interactions between the azobenzene and the biopolymer (Fig. 3).

The behavior of Azo-3N is typical for molecules intercalating between the DNA base pairs and the extent of the hypochromism is related to the strength of the intercalative interaction^41^,^42^,^43^,^44^. Such phenomenon can be expected in regard of the aromatic rings of the azobenzene core, which, by increasing the planarity of the system, provide a greater possibility to accept the guest-molecules inside the base pairs of DNA.

The π-π^*^ transition band of Azo-3N in *trans* conformation in the presence of DNA exhibits 27% hypochromicity and a 2 nm bathochromic shift. On the other hand, the *cis* isomer, under the same experimental conditions, displays 11% hypochromicity and a red shift of 6 nm. Further addition of DNA does not result in any additional changes of the absorption spectrum of both isomers. The differences in DNA binding of Azo-2N and Azo-3N seem to be strictly connected to the different number of charges along the polyamine backbone which confer to the bicationic Azo-3N a major affinity for the duplex^17^,^45^.

Exploiting the absorption changes arising upon DNA addition to Azo-3N it was possible to compare quantitatively the binding affinity of Azo-3N in both its isomeric forms toward double-stranded DNA. The binding constants were found to be 8.8 × 10^4^ M^−1^ and 4.3 × 10^3^ M^−1^ for the *trans* and *cis* form, respectively (See SI p. S7 for details). Such values agree well with those reported for a variety of intercalators bound to *ds*-DNA^46^,^47^,^48^. The differences in the magnitude of the binding constant can be directly related to the changes in shape occurring on the photo-isomerization. The planarity of the *trans* form makes it suitable as a DNA intercalator, whereas the non-planarity of the bent *cis* form decreases to some extent its intercalative ability resulting in a possible end-stacking mode^49^. Furthermore, the larger molecular size of the *cis* form acts as a steric hindrance for its entering between the base pairs, which may be an additional explanation of the differences in the DNA binding affinity.

**Figure 4:**
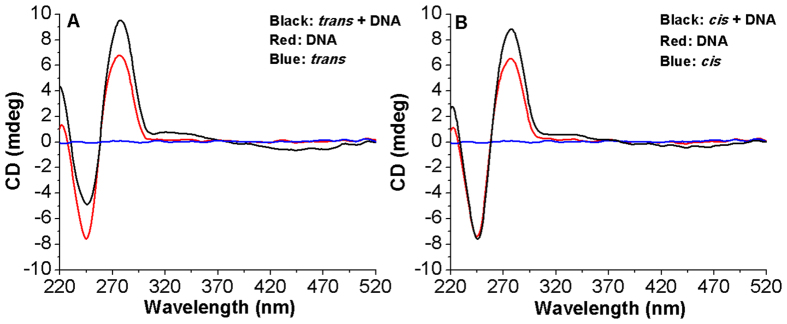
Circular dichroism spectra of *ds*-DNA in absence (red line) and in presence of Azo-3N in *trans* (**A**) (black line) and *cis* (**B**) conformation (black line). The optical inactivity of Azo-3N is shown by the blue line. The concentrations of Azo-3N and DNA were 5 × 10^−5^ M in 10 mM sodium cacodylate trihydrate (pH 7.25).

**Figure 5:**
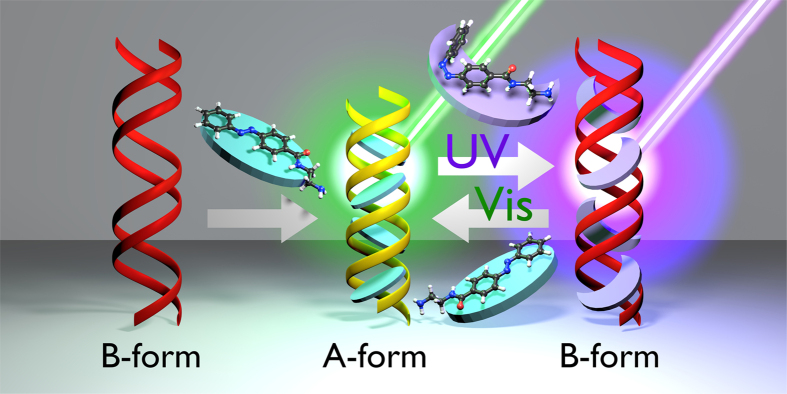
Cartoon representing the possible mechanism of interaction between Azo-3N, in both its isomeric forms, and the DNA template.

In order to elucidate the ability of Azo-3N to induce conformational changes in the DNA secondary structure, circular dichroism (CD) spectra were studied (Fig. 4). The canonical B-DNA form is characterized by two CD bands of equal intensity^50^. The positive band at ~275 nm occurs due to base stacking and the negative band at ~245 nm is attributable to the helical structure that provides asymmetric environment for the bases. Azo-3N is achiral and, hence, optically inactive. However, upon association with DNA, a bisignate induced circular dichroic band (ICD) appears for both its isomeric forms (See SI p. S9 for details). The appearance of the ICD signals in the region between 310 and 490 nm can be attributed to the coupling of the electric transition moment of the azobenzene derivative and those of the chirally arranged DNA base pairs. Intercalators usually display a weak negative or bisignate ICD signal whereas larger positive ICD signals are attributable to the groove-binding geometry^51^. However, such bands are very sensitive to the strength and orientation of the transition moment with respect to the DNA binding site. As expected, the signal magnitude of the ICD band of the *trans* isomer results increased to some extent in respect to the bent *cis* form, which is a result of the different binding strength toward the DNA template arising from higher planarity of the former. Such results can be directly related to the transition moment which, in the elongated *trans* form, should be oriented along the molecule long axis allowing a suitable penetration among the center of the intercalation sites, whereas the bent *cis* form can be laterally displaced to some extent from the binding sites resulting in a weakening of the ICD signal^51^. In order to better elucidate the binding mode, the intrinsic positive and negative bands of the B-DNA form, which are highly sensitive towards the interaction with guest molecules, were analyzed^51^. An increase of the positive band was observed upon interaction with both the *trans* and *cis* forms. On the other hand, a different trend was observed for the *trans* and *cis* isomers in affecting the DNA negative band. Binding of the *trans* form resulted in a decrease (shifting towards zero) of the CD band at 245 nm whereas the *cis* form seemed to not cause any appreciable change. The conformational changes induced by the *trans* isomer on the DNA moiety are indicative of a B-to-A DNA transition. The substantial difference in affecting the negative DNA band, for the *trans* and *cis* isomer, seems to be related to the fact that the reduced free volume of the planar *trans* form allows the azobenzene core an easy transition among the DNA bases resulting in a strong perturbation of the helicity. The larger molecular size of the bent, non-planar *cis* form hinders it from coming back to the stacked position and no obvious deformation of the helical structure is observed (See SI p. S9 for details concerning the reversibility of the process)^52^. A representation of the DNA morphology change, occurring upon interaction with the photochrome in both its isomeric forms, is proposed in Fig. 5.

Additional investigations were carried out by FT-IR spectroscopy which is a useful tool to monitor the structural changes generated by the guest molecules in the DNA structure as it provides insights into the sites involved in the interaction (see Supporting Information SI, p. S11 for details). FT-IR spectra indicate that both the phosphate groups and the DNA bases are involved in interaction with Azo-3N. In particular, the insertion of the planar conformer among the base pairs affects the B-DNA’s marker bands providing evidence of the B-to-A transition.

## Conclusion

In summary, the DNA-binding properties of two water-soluble 4-(phenylazo)benzoic acid derivatives (Azo-2N and Azo-3N) have been comprehensively investigated. The data obtained by the UV-Vis study indicate the intercalation of the Azo-3N moiety among the DNA base pairs. A substantial difference in binding strength between the bicationic Azo-3N and the monocationic Azo-2N for the duplex was observed suggesting that the number of positive charges along the photosensitive polyamines chain exerts a key role to stabilize the Azo-DNA complex. The observed different DNA-binding properties of the two isomeric forms lead to the conclusion that the elongated planar *trans* isomer induces major perturbation on the DNA moiety as compared to the bent non-planar *cis* form. Therefore, as the transition between the two forms can be accomplished photochromically, the azobenzene DNA-binding can also be directed by light. The circular dichroism data clearly point out that the conformational changes induced by the *trans* form to the *ds*-DNA are indicative of a B-to-A transition, whereas the larger molecular size of the bent non-planar *cis* form seems to not cause any significant deformation of the helical structure. Furthermore, modulation of the canonical DNA band was achieved by photochemical switching the state of the non-covalently attached photochrome which provides an example of direct regulation of the DNA secondary structure by a specific small molecule.

## Methods

### Synthesis

**Figure 6:**
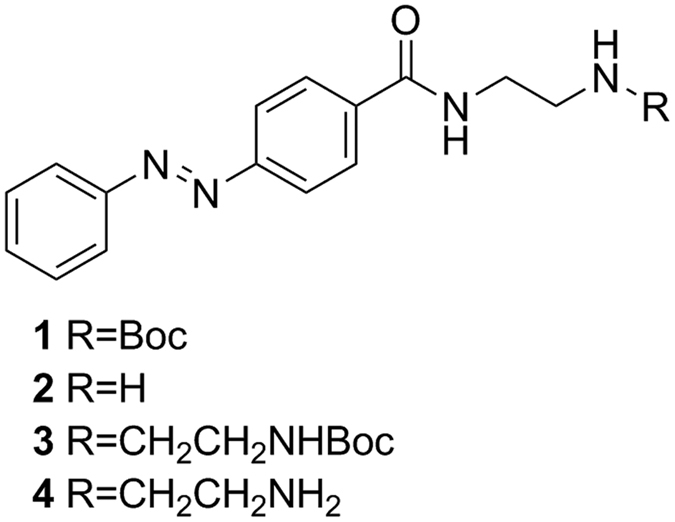
Synthetic route of the azobenzene derivatives.

The synthetic route of the azobenzene derivatives is depicted in Fig. 6.

#### Synthesis of 1

To dry dichloromethane (10 mL) solution of (2-Amino-ethyl)-carbamic acid tert-butyl ester (0.48 g, 3 mmol) and triethylamine (0.84 mL, 6 mmol), 4-(Phenylazo)benzoyl chloride was added (0.73 g, 3 mmol) and the reaction mixture was stirred overnight. The solvent was evaporated under reduced pressure and the product was purified with column chromatography on silica using gradient elution from DCM to 10%MeOH/DCM to afford **1** as orange powder (0.88 g, 80%).

^**1**^**H NMR** (600 MHz, CDCl_3_) δ 8.01–7.92 (m, 6 H), 7.56–7.48 (3 H,m), 7.37 (bs,1 H), 4.99 (bs,1 H), 3.59 (dd, ^3^J_H,H_ = 10.5, 4.9 Hz, 2 H), 3.47–3.41 (m, 2H), 1.44 (s, 9 H). ^**13**^**C NMR** (150 MHz, CDCl_3_) δ: 28.07, 39.59, 42.11, 80.22, 122.9, 128.03, 129.1, 131.65, 136.03, 152.67, 154.32, 156.44, 157.84, 167.25. **HRMS** m/z (ESI): calcd for C_20_H_24_N_4_O_3_ 391.1746 [M + Na]^+^ found 391.11759.

#### Synthesis of **2** (Azo-2N)

Compound **1** (70 mg, 0.24 mmol) was dissolved in DCM (10 mL) and TFA (150 μL) was added then stirred overnight. Solvent was removed under reduced pressure and obtained orange residue was dissolved in DCM (15 mL) and washed with saturated NaHCO_3_ solution (3 × 15 mL). The organic layer was dried over MgSO_4_, filtrated, and evaporated under reduced pressure affording **2** as orange solid (40 mg, 88%).

^**1**^**H NMR** (600 MHz, MeOD) δ 2.87 (t, ^3^*J*_H,H_ = 6.36 Hz, 2 H), 3.39 (t, ^3^*J*_H,H_ = 6.36 Hz, 2 H), 7.49–7.42 (m, 2 H), 7.83 (d, ^3^*J*_H,H_ = 7.11 Hz, 2 H), 7.87 (d, ^3^*J*_H,H_ = 8.45 Hz, 2 H), 7.92 (d, ^3^*J*_H,H_ = 8.45 Hz, 2 H). ^**13**^**C NMR** (150 MHz, MeOD) δ 40.55, 42.29, 122.3, 122.65, 128.10, 129.0, 131.5, 136.24, 152.53, 154.22, 168.4. **HRMS** m/z (ESI): calcd for C_15_H_16_N_4_O [M + H]^+^ 269.1402, found 269.1397.

#### Synthesis of **3**

To dry dichloromethane solution of 1,4-Bis-boc-1,4,7-triazaheptane (0.44 g, 1.47 mmol) and triethylamine (0.42 mL, 3 mmol), 4-(Phenylazo)benzoyl chloride was added (0.3 g, 1.22  mmol) and the reaction mixture was stirred overnight. The solvent was evaporated under reduced pressure and after purification with column chromatography over silica using 10%MeOH/DCM as eluent gave **3** as orange powder (0.45 g,72%).

^**1**^**H NMR** (601 MHz, CDCl_3_) δ 8.07–7.83 (m, 5 H), 7.61–7.41 (m, 3 H), 4.90 (s, 1 H) 3.67–3.51 (m, 4 H), 3.38–3.18 (m, 4 H), 1.56–1.28 (m, 18 H). ^**13**^**C NMR** (150 MHz, CDCl_3_),28.50, 28.45, 39.52, 40.82, 46.65, 47.84, 79.5, 81.0, 122.88, 123.14, 128.2, 129.25, 131.6 136.0, 152.66, 154.26, 156.12, 157.58, 166.96. **HRMS** m/z (ESI): calcd for C_27_H_37_N_5_O_5_ [M + H]^+^ 512.2873, found 512.2874.

#### Synthesis of **4** (Azo-3N)

**3** (0.2 g, 0.39 mmol) was dissolved in DCM (5 mL) and TFA (300 μl) was added then was stirred for 12 hours. Solvent was removed under reduced pressure resulting in **4** as orange solid (0.19 g, 90%).

^**1**^**H NMR** (600 MHz,D_2_O) δ: 8.02 (d, ^3^*J*_H,H_ = 8.25 Hz, 2 H), 7.96 (m, 2 H), 7.91–7.95 (m, 2 H), 7.67-7.62 (m, 3 H), 3.83 (t, ^3^*J*_H,H_ = 5.67 Hz, 2 H), 3.56-3.42 (m, 6 H). ^**13**^**C NMR** (150 MHz, D_2_O) δ 35.47,36.48, 44.46, 48.11, 122.57, 122.67, 128.63, 129.63, 132.37, 134.80, 154.17, 151.97,170.46. **HRMS** m/z (ESI): calcd for C_17_H_21_N_5_O [M + H]^+^ 312.1824. found 312.1827.

### Apparatus

NMR-spectra were recorded on a Bruker AvanceTM 600 MHz spectrometer. Mass spectra were conducted with a WATERS LCT Premier XE mass spectrometer (ESI). The UV-Vis absorption spectra were recorded on a Perkin Elmer Lambda 20 UV-Vis spectrometer. Circular dichroism spectra were recorded with a Jasco J-815 spectropolarimeter (Jasco Inc, USA) equipped with the Jasco Peltier-type temperature controller (CDF-426S/15). All measurements were carried out in 1.0 cm path length quartz cells. The infrared spectra were collected, on the diamond crystal surface under vacuum (<1 hPa), using a Bruker Vertex70v FT-IR spectrometer. A Metrohm 902 Titrando digital pH meter, equipped with Tiamo 2.3 software, was used to detect the pH values of the solutions.

### Reagents and preparation of stock solutions

Common reagent-grade chemicals obtained from commercial suppliers were used without further purifications. The stock solution of deoxyribonucleic acid sodium salt from salmon testes (DNA), purchased from Sigma Aldrich Chem. Co., was prepared by dissolving an appropriate amount of solid DNA powder in 10 mM sodium cacodylate buffer (pH 7.25). Stock solution was stored at 4 °C for 24 hours with occasional stirring and was used after no more than for 3 days. The appropriate DNA solution concentrations were determined by absorption spectrometry according to the absorbance at 260 nm. The Azo-2N and Azo-3N stock solutions were prepared by dissolving appropriate amounts of the azobenzene derivatives in double distilled water to a final concentration of 0.56 and 0.32 mM, respectively. The stock solutions were stored protected from light by wrapping the vials with aluminum foil. The Azo-3N *trans*-to-*cis* ratio was assessed by NMR spectroscopy according to the intensity ratios of the corresponding signals (data not shown) which provide the following values: before UV light illumination *trans* = 78% and *cis* = 22%; after UV light illumination (313 nm) *trans* = 25% and *cis* = 75%.

### UV-Vis measurements

The UV-Vis absorption spectra were recorded at 298 K keeping the concentration of the azobenzene derivatives constant and adding incremental amounts of DNA. After addition of DNA to the azobenzene solutions, the resulting system was subjected to UV-Vis analysis in the 200–800 nm range. The decrease of the absorbance was monitored upon increments in DNA concentration. In order to resolve the contribution from exclusively Azo-2N and Azo-3N the spectrum of the equimolar solution of DNA added has been subtracted in the full measured range. The photoinduced isomerization reactions of Azo-2N and Azo-3N was performed by using a high pressure Hg Oriel lamp equipped with interference filters at 313 and 436 nm. The resulting light power density was 0.41 mW/cm^2^ (*trans*-*cis*) and 1.04 mW/cm^2^ (*cis*-*trans*). See Supporting Information SI, p. S8 for details concerning the corrected absorbance variation.

### Circular dichroism measurements

CD spectra were recorded at 298 K in the wavelength range of 200–800 nm at different azobenzene/DNA molar ratios and keeping constant the DNA concentration. Before use, the optical chamber of the CD spectrometer was deoxygenated with dry nitrogen and was held in a nitrogen atmosphere during the measurements. Each spectrum was averaged from five successive accumulations and the cacodylate buffer contribution has been subtracted. The time required to record the CD spectra does not affect the *trans*:*cis* ratio. Such evidence was checked by measuring and monitoring the variation of the absorbances of samples exposed to the UV beam used for CD measurement upon five accumulations (see SI, p. S11 for details).

### FT-IR spectroscopic measurements

The infrared spectra were recorded, after incubation of the salmon sperm DNA/azobenzene solution, via the Attenuated Total Reflection (ATR) method, in the spectral range 2000–400 cm^−1^ with a resolution of 4 cm^−1^ and accumulation of 64 scans, and transformed into absorbance spectra using OPUS software. Spectra subtraction [(*ds*-DNA solution + Azo-3N)–Azo-3N solution] was performed to make sure that the observed changes in the DNA shift peak position were attributable exclusively to ligand interactions.

### Binding constant

The intrinsic association constant for Azo-3N in both its conformations towards *ds*-DNA was calculated by using the Benesi-Hildebrand^53^ (K_a_) and Scatchard^54^ (K_b_) equations:


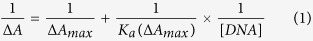



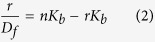


where ΔA is the difference between the absorbance of Azo-3N in the absence and presence of DNA, ΔA_max_ is the final absorbance of the Azo-DNA adduct which indicates saturation of interaction, r is the mole of ligand bound per mole of macromolecule and D_f_ is the molar concentration of the free Azo-3N. The calculated binding constants take into account the *trans*:*cis* ratio.

## Additional Information

**How to cite this article**: Deiana, M. *et al*. Photochromic switching of the DNA helicity induced by azobenzene derivatives. *Sci. Rep.* **6**, 28605; doi: 10.1038/srep28605 (2016).

## Supplementary Material

Supplementary Information

## Figures and Tables

**Figure 1:**
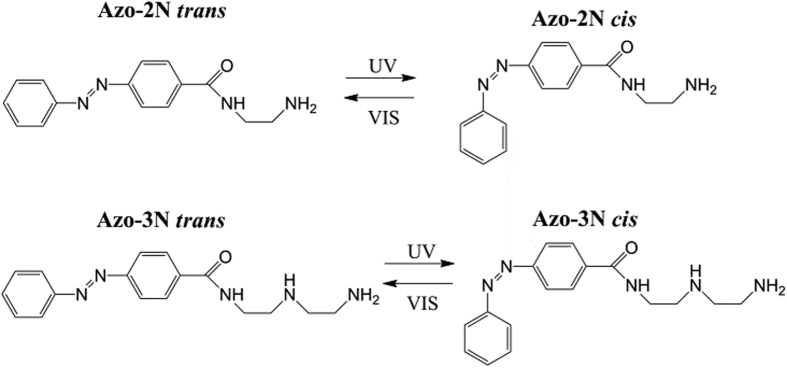
Structures of the photochromic azobenzene derivatives Azo-2N and Azo-3N. Interconversion pathways between the relevant forms of the azobenzene photoswitch.
