# Supplementary material for: Photochromic switching of the DNA helicity induced by azobenzene derivatives
Source: Sci Rep. 2016 Jun 24;6:28605. doi: 10.1038/srep28605 (PMC4919647; doi:10.1038/srep28605)
Supplement: Supplementary Information [file srep28605-s1.pdf]

Supplementary Information

Photochromic switching of the DNA helicity induced by azobenzene derivatives

Marco Deiana[1], Ziemowit Pokladek[2], Joanna Olesiak-Banska[1], Piotr Mlynarz[2], Marek Samoc[1] and Katarzyna Matczyszyn[1]*

[1]*Advanced Materials Engineering and Modelling Group, Faculty of Chemistry, Wroclaw University of Science and Technology, Wyb. Wyspianskiego 27, 50-370 Wroclaw (Poland)*

[2] *Department of Bioorganic Chemistry, Faculty of Chemistry, Wroclaw University of Science and Technology, Wyb. Wyspianskiego 27, 50-370 Wroclaw (Poland)*

*Correspondence to katarzyna.matczyszyn@pwr.edu.pl

Table of Contents:

- Computations p. S2
- Binding constant p. S7
- Irradiation cycle p. S9
- Assessing the absence of aggregation p. S9
- Influence of the UV beam used to record the CD spectra p. S11
- FT-IR p. S11
- References p. S16

Computations

Calculations were carried out by Gaussian 09 package[1], M062X functional with 6-31++G(d,p) basic set was used.[2] SCRF PCM model of solvent was used with water as a solvent, all structures were confirmed as stationary points by frequency analysis.[3] We used fully protonated polyamino chain, which corresponds to the state of molecule at physiological pH. The dihedral angle between phenyl ring of azobenzene and amide group was found to be around 27 degrees in both *trans* and *cis* forms.

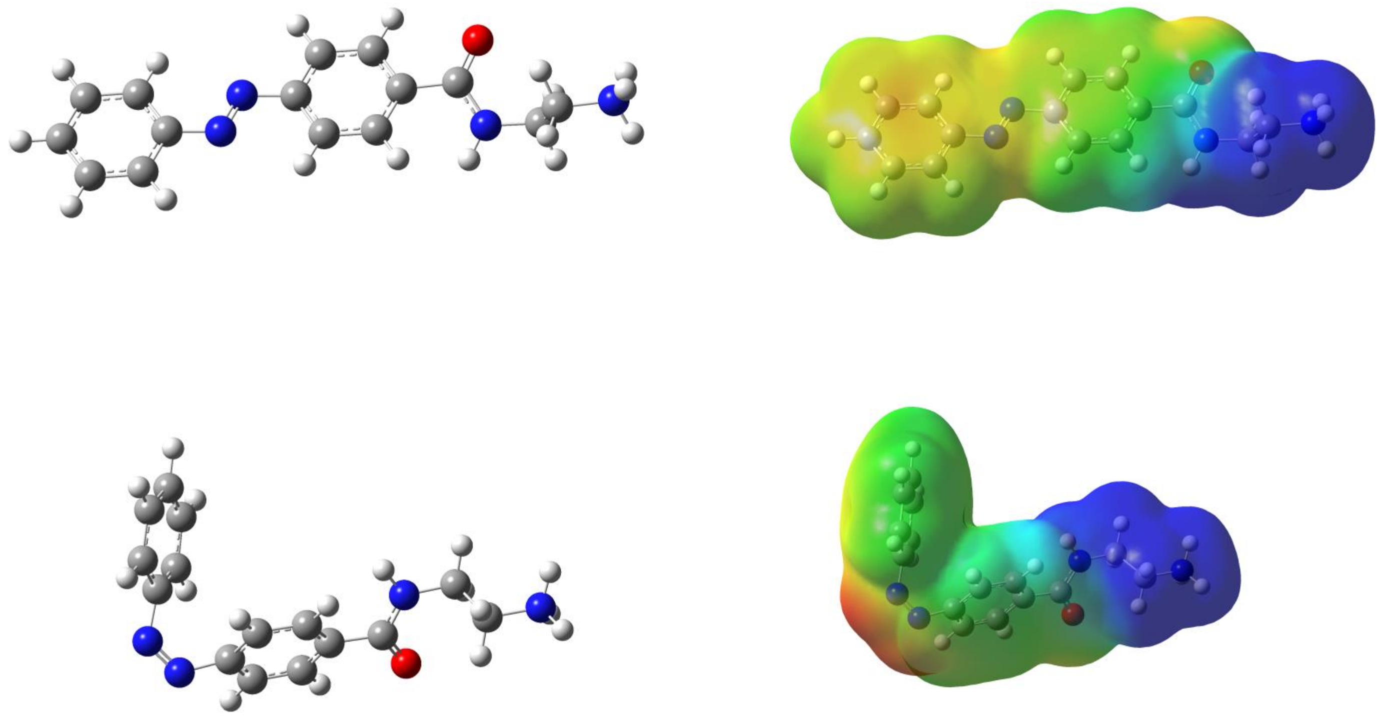

Figure S1. Optimized *trans* and *cis* structures (left) and corresponding electrostatic potential (right) for **2** (Azo-2N).

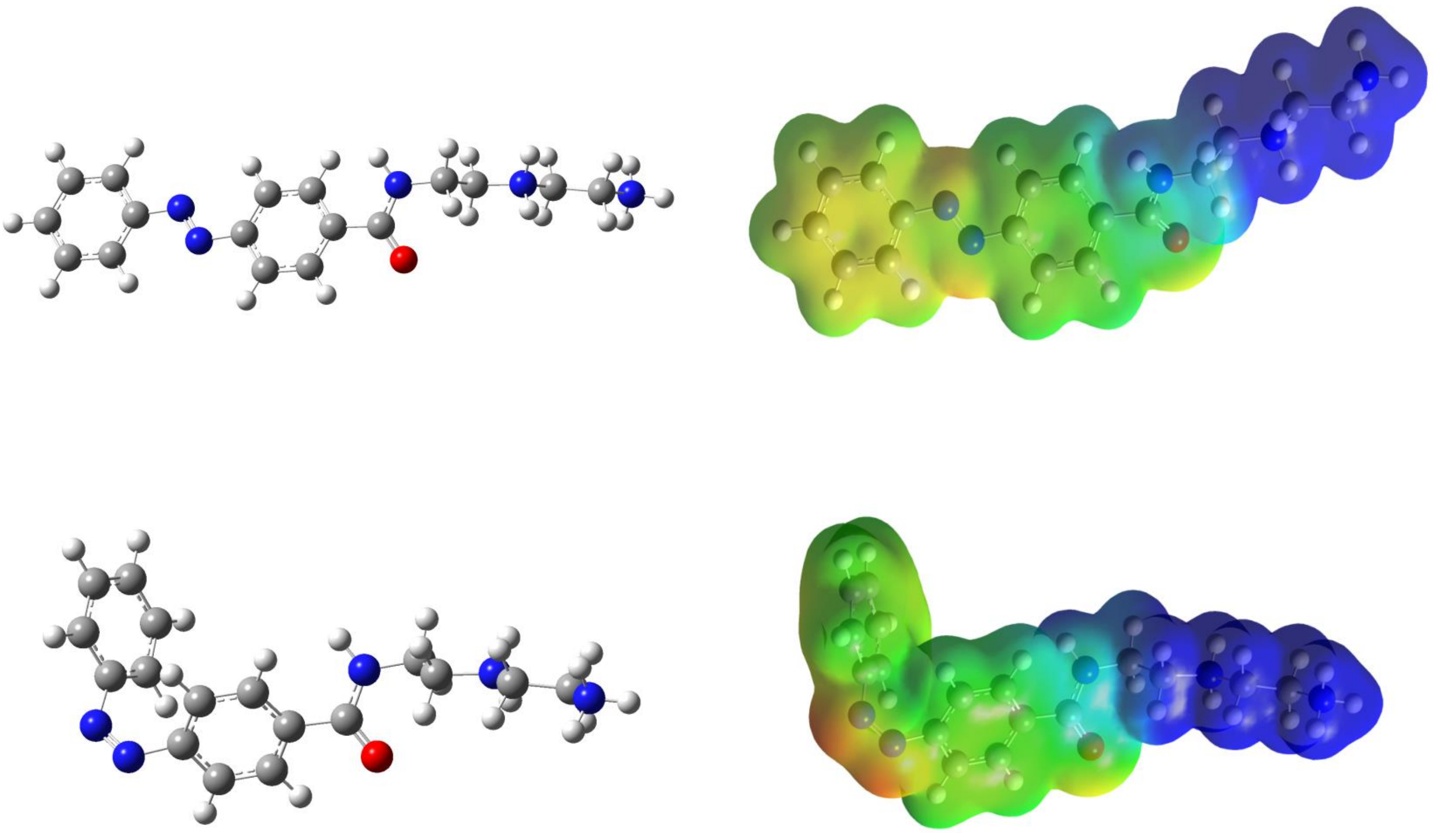

Figure S2. Optimized *trans* and *cis* structures (left) and corresponding electrostatic potential (right) for **4** (Azo-3N).

Cartesian coordinates of *trans* form of **2** (Azo-2N).

C	6.41255800	-1.55147100	-0.10371600
C	5.01973100	-1.54090100	-0.05895300
C	4.33283700	-0.32541900	-0.03947000
C	5.03273400	0.88840300	-0.06530700
C	6.42124000	0.86946100	-0.11013500
C	7.11311100	-0.34677100	-0.12904100
H	6.94744300	-2.49534700	-0.11908100
H	4.44893800	-2.46425700	-0.03906700
H	4.48548500	1.82399300	-0.05078900
H	6.97172100	1.80463100	-0.13084500
N	2.91286100	-0.43231900	0.00588700
N	2.30156400	0.65101800	0.02569000
C	0.88026800	0.53159200	0.06802400
C	0.19389300	-0.68708900	0.12850500
C	0.17741000	1.73764700	0.03696200
C	-1.19385300	-0.68718900	0.16165100
H	0.74916500	-1.61744200	0.14065000
C	-1.21273200	1.73170400	0.05739100

H	0.73371000	2.66848100	-0.00928900
C	-1.90377000	0.52093100	0.12960900
H	-1.71786500	-1.63818100	0.17989500
H	-1.77348300	2.65955500	0.02078400
C	-3.40192900	0.57474400	0.13269900
O	-4.01460500	1.50838700	-0.38438200
N	-4.05097700	-0.45465600	0.73097900
C	-5.49804700	-0.48584400	0.77265600
H	-5.87071100	0.51350400	1.02000700
H	-5.80620800	-1.18101700	1.55567000
H	8.19791800	-0.35043900	-0.16428300
H	-3.53330300	-1.15486700	1.24126200
C	-6.03512900	-0.92300200	-0.58847700
H	-5.74860500	-1.94865300	-0.81934000
H	-5.68216500	-0.25303700	-1.37242000
N	-7.53300800	-0.86638200	-0.60434700
H	-7.90183300	-1.14482400	-1.51802900
H	-7.94750700	-1.49252100	0.09205200
H	-7.87886700	0.07891500	-0.41397200

Cartesian coordinates of *cis* form of **2** (Azo-2N).

N	3.80768900	-1.38942700	-0.61825500
N	2.80903500	-2.12412900	-0.63324300
C	1.48829200	-1.62734600	-0.35130300
C	0.93091000	-0.58464500	-1.09453200
C	0.72071000	-2.33289200	0.57572300
C	-0.38946700	-0.21500400	-0.86548400
H	1.52025600	-0.07524900	-1.85013000
C	-0.59173700	-1.94394800	0.81324700
H	1.16266300	-3.16693900	1.11178200
C	-1.15072300	-0.87797400	0.10305100
H	-0.82220000	0.57616100	-1.47038500
H	-1.19923700	-2.46761300	1.54381100
C	-2.57628500	-0.51827000	0.39209200
O	-3.37097300	-1.33723000	0.85266400
C	3.74318600	-0.02358900	-0.16721800
C	4.33499500	0.94083600	-0.98218100
C	3.24065100	0.31209300	1.09247300
C	4.35574700	2.26990400	-0.56492300
H	4.75537000	0.64512500	-1.93871900
C	3.29773600	1.63775800	1.51444900
H	2.81997700	-0.45465200	1.73559800
C	3.83910700	2.61994700	0.68306700
H	4.78998500	3.02810700	-1.20844600
H	2.91655600	1.90402200	2.49507300
H	3.87106800	3.65289400	1.01390500
N	-2.94919600	0.75845100	0.12501200
C	-4.30281600	1.20083800	0.38353400
H	-4.30342400	2.29096900	0.43960800
H	-4.63447700	0.79768400	1.34581900
H	-2.25894900	1.44330000	-0.14547900

C	-5.22520500	0.71348800	-0.73107700
H	-4.94668300	1.13931800	-1.69472800
H	-5.21694200	-0.37494900	-0.78954300
N	-6.63795400	1.13104200	-0.45284600
H	-7.27281100	0.79405500	-1.18183100
H	-6.97624600	0.75671100	0.43881600
H	-6.73473200	2.15016100	-0.41666100

Cartesian coordinates of *trans* form of **4** (Azo-3N).

C	7.71524300	-1.83569900	0.27032300
C	6.34063000	-1.66913200	0.42694200
C	5.71812800	-0.51898300	-0.06221100
C	6.46542600	0.47362400	-0.71058100
C	7.83542800	0.30005100	-0.86287800
C	8.46269400	-0.85166900	-0.37443800
H	8.19913000	-2.72961000	0.64985100
H	5.73500100	-2.42060500	0.92435000
H	5.96878400	1.36110500	-1.08594300
H	8.42115000	1.06374000	-1.36461800
N	4.31060100	-0.45578300	0.14795000
N	3.75694600	0.57055500	-0.28532100
C	2.34622100	0.61773500	-0.07610200
C	1.60679600	-0.40083000	0.53708900
C	1.70997200	1.76941900	-0.54314300
C	0.23470300	-0.25309200	0.68531300
H	2.10856100	-1.29774100	0.88076000
C	0.33407300	1.90856600	-0.40070500
H	2.30532700	2.54291600	-1.01785100
C	-0.40793600	0.90326200	0.22197500
H	-0.33277000	-1.06256700	1.13470500
H	-0.17629100	2.79285900	-0.76734800
C	-1.88970100	1.09946300	0.33029200
O	-2.50675900	1.81549200	-0.45758600
N	-2.52381900	0.44822300	1.33753800
C	-3.95582200	0.57691100	1.50590600
H	-4.24001300	1.62602000	1.37485100
H	-4.21427000	0.26292800	2.51823600
H	9.53353100	-0.97795200	-0.49932800
H	-1.99400400	-0.04852700	2.03841100
C	-4.65641900	-0.29361800	0.46355800
H	-4.45486600	-1.35246800	0.62966300
H	-4.34260600	-0.00581900	-0.54135100
N	-6.14949500	-0.11344700	0.51896500
H	-6.48731500	-0.33507500	1.46326400
H	-6.37247400	0.87617600	0.35785000
C	-6.85307100	-0.97125700	-0.48492700
H	-6.65885400	-2.00797700	-0.20516200
H	-6.39353800	-0.76641500	-1.45303800
C	-8.34417800	-0.65452900	-0.49624500
H	-8.81147000	-0.84478000	0.47000400

H	-8.53492700	0.37606700	-0.79586100
N	-9.03727100	-1.52799600	-1.49195900
H	-8.97325900	-2.52253800	-1.25074600
H	-10.03472600	-1.29507900	-1.52970400
H	-8.66788200	-1.41078700	-2.44152400

Cartesian coordinates of *cis* form of **4** (Azo-3N).

N	-5.12384000	-1.30514500	0.88729300
N	-4.14350800	-2.06262600	0.83476700
C	-2.84380200	-1.60625500	0.41940700
C	-2.19959900	-0.55397900	1.07446800
C	-2.17858700	-2.35710200	-0.55007900
C	-0.89940400	-0.21944200	0.71280900
H	-2.70723500	-0.00917900	1.86395000
C	-0.88578000	-2.00600700	-0.91778800
H	-2.68330000	-3.19652300	-1.01773600
C	-0.24327300	-0.93085500	-0.29730200
H	-0.39764200	0.58186200	1.24680500
H	-0.35805000	-2.56580800	-1.68264600
C	1.15844900	-0.62227800	-0.72368400
O	1.89708400	-1.48661500	-1.19497300
C	-5.07342600	0.04598500	0.39297900
C	-5.58507800	1.04114600	1.22533200
C	-4.66555100	0.34152100	-0.91036400
C	-5.61728300	2.35956600	0.77629600
H	-5.93419200	0.77732500	2.21905600
C	-4.73397400	1.65703600	-1.36117400
H	-4.30929100	-0.44855300	-1.56399800
C	-5.19340200	2.66927300	-0.51640800
H	-5.98875400	3.14123300	1.43090500
H	-4.42687700	1.89147400	-2.37532100
H	-5.23503800	3.69391000	-0.87109400
N	1.58148500	0.65809300	-0.56957300
C	2.91960000	1.03380100	-0.97410700
H	2.95452300	2.11692700	-1.09797500
H	3.14733200	0.56020400	-1.93475900
H	0.93322000	1.38059000	-0.29274500
C	3.91217100	0.57032000	0.09195700
H	3.80951500	1.14700900	1.01195500
H	3.77792100	-0.49241900	0.29931300
N	5.32896400	0.74009300	-0.38552100
H	5.47059200	0.14395900	-1.20938000
H	5.47384700	1.70648300	-0.70264500
C	6.33037500	0.39846100	0.67140800
H	6.22461000	1.14330300	1.46189100
H	6.05739100	-0.58399900	1.06033800
C	7.73407600	0.39786600	0.07788300
H	8.01047300	1.37687900	-0.31360700
H	7.84216300	-0.35224500	-0.70580200
N	8.72662100	0.06029900	1.14302100
H	9.67519800	0.04908900	0.75452000

H	8.72447900	0.74671500	1.90464000
H	8.56133000	-0.86465000	1.55442300

Binding constant

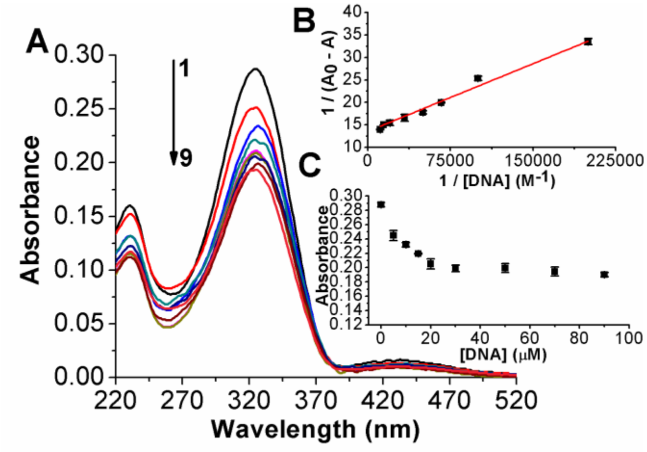

Figure S3. (**A**) Absorption spectra of Azo-3N *trans* (2×10^{-5} M) treated with: 0, 5, 10, 15, 20, 30, 50, 70 and 90 µM (curves 1-9) of DNA in 10 mM sodium cacodylate trihydrate (pH 7.25). (**B**) Benesi-Hildebrand plot. A_0 and A are the corrected absorbance values of the azobenzene derivative in absence as well as in presence of DNA, respectively and take into account the *trans*:*cis* ratio. (**C**) Plot of the absorbance variation as a function of the DNA concentration including the contribution of the mixed *trans* and *cis* isomers. [DNA] = 0, 5, 10, 15, 20, 30, 50, 70 and 90 µM.

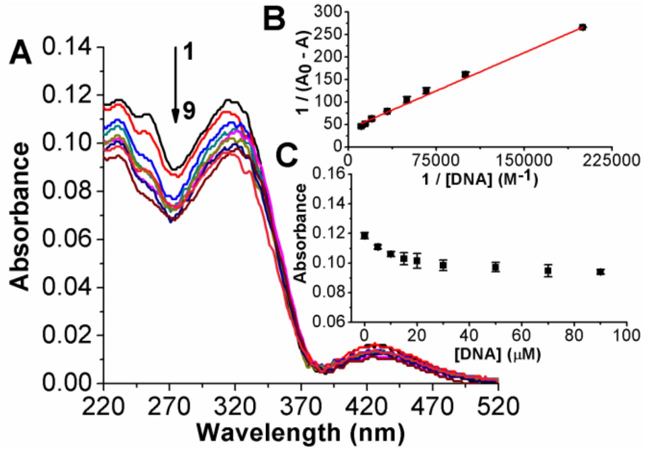

Figure S4. (**A**) Absorption spectra of Azo-3N *cis* (2×10^{-5} M) treated with: 0, 5, 10, 15, 20, 30, 50, 70 and 90 µM (curves 1-9) of DNA in 10 mM sodium cacodylate trihydrate (pH 7.25). (**B**) Benesi-Hildebrand plot. A_0 and A are the corrected absorbance values of the azobenzene derivative in absence as

well as in presence of DNA, respectively and take into account the *trans*:*cis* ratio. (**C**) Plot of the absorbance variation as a function of the DNA concentration including the contribution of the mixed *trans* and *cis* isomers. [DNA] = 0, 5, 10, 15, 20, 30, 50, 70 and 90 µM.

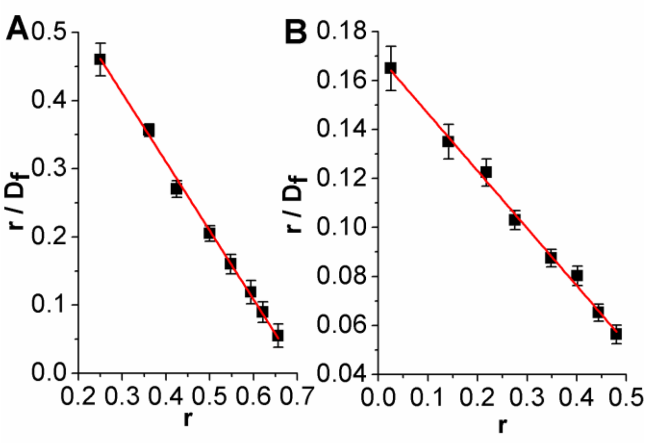

Figure S5. A) Scatchard plot for the binding of Azo-3N *trans* taking into account the *trans*:*cis* ratio. r is the moles of ligand bound per mole of nucleic acid and D_f is the molar concentration of the free ligand. **B)** Scatchard plot for the binding of Azo-3N *cis* taking into account the *trans*:*cis* ratio. r is the moles of ligand bound per mole of nucleic acid and D_f is the molar concentration of the free ligand.

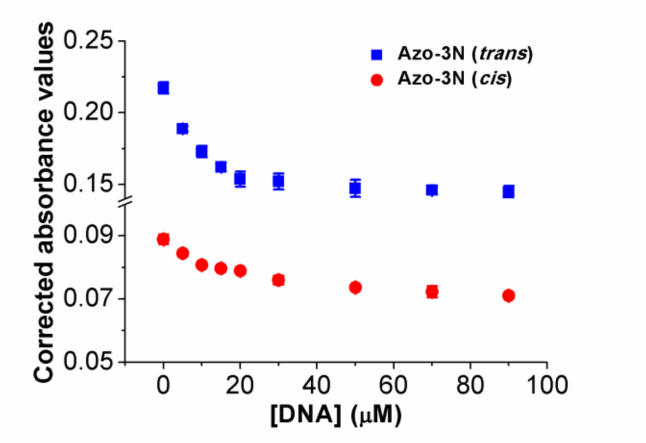

Figure S6. Plot of the corrected absorbance variation as a function of the DNA concentration taking into account the *trans*:*cis* ratio. [DNA] = 0, 5, 10, 15, 20, 30, 50, 70 and 90 µM.

Binding data analysis provided the following values of the association constant:

K_a (DNA-Azo-3N *trans*) = 8.8 ± 0.2 × 10^4 M^{-1}

K_a (DNA-Azo-3N *cis)* = 4.3 ± 0.6 × 10^3 M^{-1}

K_b (DNA-Azo-3N *trans*) = $9.0 \pm 0.6 \times 10^4$ M^{-1}

K_b (DNA-Azo-3N *cis*) = $4.6 \pm 0.7 \times 10^3$ M^{-1}

Irradiation cycles

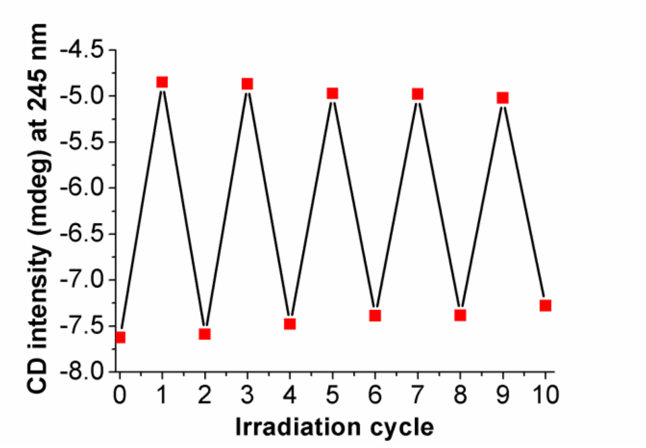

Figure S7. Plot of the reversible change of the monitored CD intensity at 245 nm under UV / Vis illumination after 10 cycles. [DNA] / [Azo-3N] = 1.

Assessing the absence of aggregation

In order to test the absence of aggregation, first we performed UV-Vis measurements at different Azo-3N (dark solution) concentrations. As can be seen from the inset of Figure S8, a linear trend between the photochrome concentration and the absorbance was observed indicating the absence of aggregate state. To further confirm our statement we also performed CD measurements keeping constant the DNA concentration and adding different amounts of Azo-3N (*trans*). As shown in Figure S8-S9, upon complexation a bisignate ICD signal in the region between 310 and 490 nm appeared and its intensity was not affected by the concentration of the photochrome. It is known that isolated monomers can be distinguished from dimers or higher order complexes by carefully looking at the shape and intensity of the ICD bands. In the case of aggregation, a nonlinear increase of the ICD signals along with change in shape of the band is usually observed. In our case the shape and intensity of the ICD signals were the

same within the whole titration range and no strong exciton CD signal and/or additional higher order splitting was found leading to the conclusion that Azo-3N bound the duplex in a monomeric fashion.

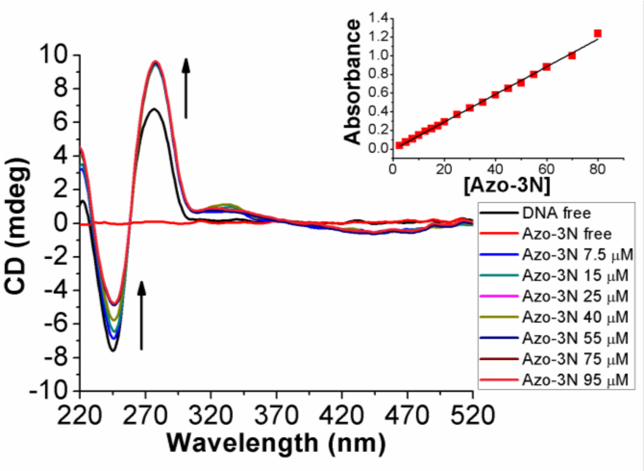

Figure S8. Circular dichroism spectra of *ds*-DNA in absence and in presence of Azo-3N in *trans* form. The optical inactivity of Azo-3N is shown by the red line. The concentration of DNA was 5 × 10^{-5} M in 10 mM sodium cacodylate trihydrate (pH 7.25). The concentration of Azo-3N was 0, 0.75, 1.5, 2.5, 4.0, 5.5, 7.5 and 9 × 10^{-5} M. **Inset:** plot of absorbance *vs.*[Azo-3N] ranging from 0.25 to 8 × 10^{-5} M.

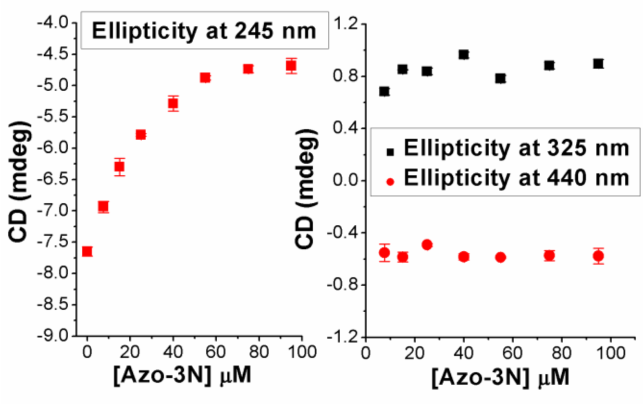

Figure S9. The comparative graphs represent the changes in DNA molar ellipticity at 245 nm and those related to the ICD signals at 325 and 440 nm.

Influence of the UV beam used to record the CD spectra

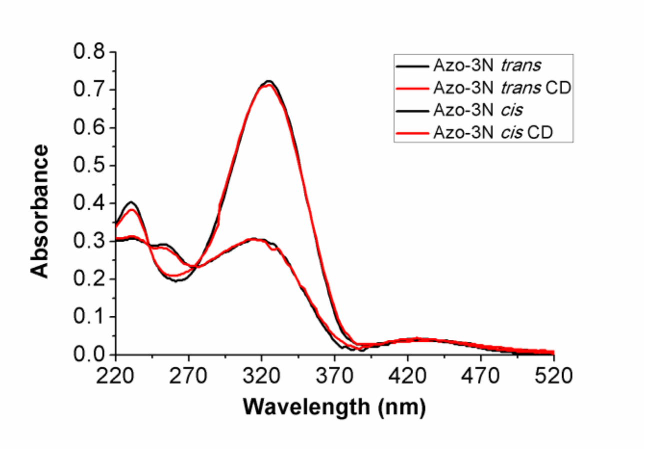

Figure S10. UV-Vis spectra of Azo-3N in both its conformations before (black lines) and after (red lines) exposure to the UV beam of the CD apparatus. The concentration of Azo-3N was 5×10^{-5} M.

Fourier transform infrared (FT-IR) spectroscopy

The FT-IR spectra of free salmon sperm DNA and the relative structural variations caused by Azo-3N in both its conformations were studied in aqueous solution (pH 7.25) at different Azo-3N/DNA molar ratios. The occurrence of the interaction can be deduced by comparing the individual DNA and the Azo-3N spectra with those of the Azo-3N-DNA complex. The FT-IR spectrum of the free DNA shown in Figure S11, is mainly constituted of bands confined in the 1800 - 400 cm^{-1} region arising from the ring vibrations of the nitrogenous bases (C=O, C=N), symmetric and asymmetric stretching vibrations of the phosphate groups (PO_2) and stretching of the phosphate-ribose diester linkage (C-O, P-O). The band at 1684 cm^{-1} is due to the in-plane stretching vibrations of guanine (G) while the band at 1653 cm^{-1} is attributable primarily to the vibrations of C_4=O of thymine (T).[4,5] The bands at 1579 and 1529 cm^{-1} can be ascribed to in-plane stretching vibrations of C8=N7 of purine ring and cytosine and guanine residues.[4,5,6] The vibrational bands at 1606, 1487 and 1417 cm^{-1} are typical of adenine (A), cytosine (C), and ring vibrations of guanine (G) bases, respectively.[5,6,7] The band at 1456 cm^{-1} is assigned to C-N glycosyl

bond.[7] The bands at 1242, 1086, 1068 and 960 cm^{-1} are related to the asymmetric and symmetric stretching vibration of phosphate group.[8] Moderate strength peaks are located at 1369, 1294, 885, 829, 783 and 729 cm^{-1} corresponding to the stretching of C-O, P-O (phosphate-ribose diester linkage) and N-H out of plane bending vibrations.[9]

The FT-IR spectra of Azo-3N in *trans* and *cis* conformation are shown in Figure S11. Both spectra are very similar and present a strong band at 1675 cm^{-1} that can be assigned to the amide C=O stretching. The bands at 1558 and 1542 cm^{-1} can be attributed to the C=C stretching vibrations of the benzene rings. The peaks at 1508 and 1496 cm^{-1} arise from the stretching of the C-N and the rocking of the C-H groups, respectively. The band at 1455 cm^{-1} can be ascribed to the stretching vibration of the methylene spacer groups. The characteristic azo (N=N) bond stretching vibration is found at 1436 cm^{-1}. The bands at 1203 and 1135 cm^{-1} are due to C-N_{azo} and C-N stretching vibrations, respectively. Additional bands are found at 840, 800, 777 and 723 cm^{-1} presumably assigned to the C-H wagging of the aromatic rings.

Azo-3N-DNA FT-IR spectra, recorded for solutions with different Azo-3N/DNA molar ratios are characterized by shifts of the bands relative to the symmetric and asymmetric vibration of phosphate group and of those related to the DNA bases (Fig. S12). In particular, in the system with a low Azo-3N(*trans*)/DNA mole ratio (1/5) the bands related to the bases at 1684 cm^{-1} (G), 1606 cm^{-1} (A), 1579 cm^{-1} (G), 1529 cm^{-1} (C), 1487 cm^{-1} (C) and 1417 cm^{-1} (G) were shifted to 1697, 1608, 1578, 1528, 1489 and 1418 cm^{-1}, respectively. When the molar ratios of Azo-3N(*trans*) to DNA increased (r=1/2.5 and r=1/1), the bands at 1684, 1606, 1487 and 1417 cm^{-1} were not further perturbed, while the bands at 1579 and 1529 cm^{-1} were shifted to 1577 and 1527 cm^{-1}, respectively. It is important to stress that no shift was observed for the band at 1653 cm^{-1} assigned to thymine for each Azo-3N(*trans*)/DNA molar ratio considered, thus ruling out its involvement in the DNA interaction.

The intercalative behavior of the azobenzene derivative can be confirmed by the direct evidence of the involvement of guanine and cytosine sequences in the association process, which, unstacking easily allow to the ligand a direct access among the DNA bases.[10]

Some of the bands related to asymmetric and symmetric stretching vibration of the phosphate groups at 1369, 1242, 1086, 1068 cm^{-1} were not perturbed at low Azo-3N(*trans*)/DNA mole ratio (1/5) while the bands at 960 and 729 cm^{-1} were shifted to 962 and 735 cm^{-1} , respectively. At higher molar ratios (r=1/2.5 and r=1/1) the bands at 1369, 1242, 1086, 1068 and 960 cm^{-1} were shifted to 1371, 1238, 1080, 1072 and 966 cm^{-1}, respectively and no further shift of the band at 735 cm^{-1} was observed. The bands at 1294 and 783 cm^{-1} attributed to sugar conformations were not affected of the presence of the ligand for each molar ratio considered. Spectral changes observed pointed out the occurrence of slight external binding interactions probably of electrostatic nature.

The B-DNA marker band at 829 cm^{-1} attributed to S-C_2 endo/anti sugar pucker-phosphodiester mode and that at 885 cm^{-1} were shifted to 825 and 887 cm^{-1} at mole ratio (r=1/5 and r=1/2.5). By increasing the Azo-3N concentration (r=1/1) no further shift of the band at 829 cm^{-1} was observed while the band at 885 cm^{-1} showed an additional shift to 889 cm^{-1}. Moreover, the band at 1456 cm^{-1}, also responsible for the B-DNA form, was shifted to 1446 cm^{-1} at mole ratio (r=1/1). Spectral features here are not indicative of a complete DNA transition from the B-to-A form but more representative of an intermediate stage in which the A-conformation is predominant.

Similarly, the Azo-3N in *cis* conformation acts as its *trans* isomer however with some remarkable differences. In particular, in the system with a low Azo-3N(*cis*)/DNA mole ratio (1/5) the bands related to the bases at 1684 cm^{-1} (G), 1606 cm^{-1} (A), 1579 cm^{-1} (G), 1529 cm^{-1} (C), 1487 cm^{-1} (C) and 1417 cm^{-1} (G) were shifted to 1685, 1608, 1577, 1527, 1489 and 1419 cm^{-1}, respectively. By increasing the concentration of the ligand (r=1/2.5 and r=1/1) no shift of the bands at 1684, 1606, 1579, 1529, 1487 and 1417 cm^{-1} was noticed while the band at 1606 cm^{-1} showed upward shift to 1610 cm^{-1} at the highest molar

ratio. As reported above for the *trans* isomer, also the *cis* conformation did not show any shift of the band at 1653 cm^{-1} for each Azo-3N(*cis*)/DNA molar ratio considered. As expected, the interaction of Azo-3N in the *cis* form seems to involve the same binding sites of the *trans* isomer, but causing smallest changes in the DNA moiety.

The asymmetric and symmetric stretching vibration of the phosphate groups at 1369, 1086, 1068 and 960 cm^{-1} were shifted to 1373, 1083, 1067 and 964 cm^{-1} at low molar ratio (r=1/5). At higher molar ratios (r=1/2.5 and r=1/1) the bands at 1242, 1086, 1068, 783 and 729 cm^{-1} were shifted to 1226, 1082, 1063, 781 and 727 cm^{-1}, respectively. The bands at 1369 and 960 cm^{-1} were not additionally modified by the increase of the ligand concentration. The band at 1294 cm^{-1} was not affected of the presence of the guest molecule for each molar ratio considered. The trend of the *cis* isomer is very similar to that depicted for the azobenzene derivative in the *trans* conformation mentioned above, indicating a possible outside binding contribution of the ligand with the phosphate-sugar backbone of the duplex.

In the system with a low Azo-3N(*cis*)/DNA mole ratio (1/5) the DNA marker bands at 829 and 885 cm^{-1} were shifted to 833 and 887 cm^{-1}. When the molar ratio was increased (r=1/2.5 and r=1/1), the band at 885 cm^{-1} further shifted to 889 cm^{-1} while the band at 829 cm^{-1} remained unchanged. Unlike the *trans* derivative, the *cis* isomer did not perturb the band at 1456 cm^{-1}, responsible for the B-conformation of DNA, at all the molar ratios considered. Such results pointed out that the bent non-planar *cis* form slightly affected the B-DNA morphology if compared to the elongated planar *trans* form. These findings are in accordance with the results found using the various spectroscopic approaches.

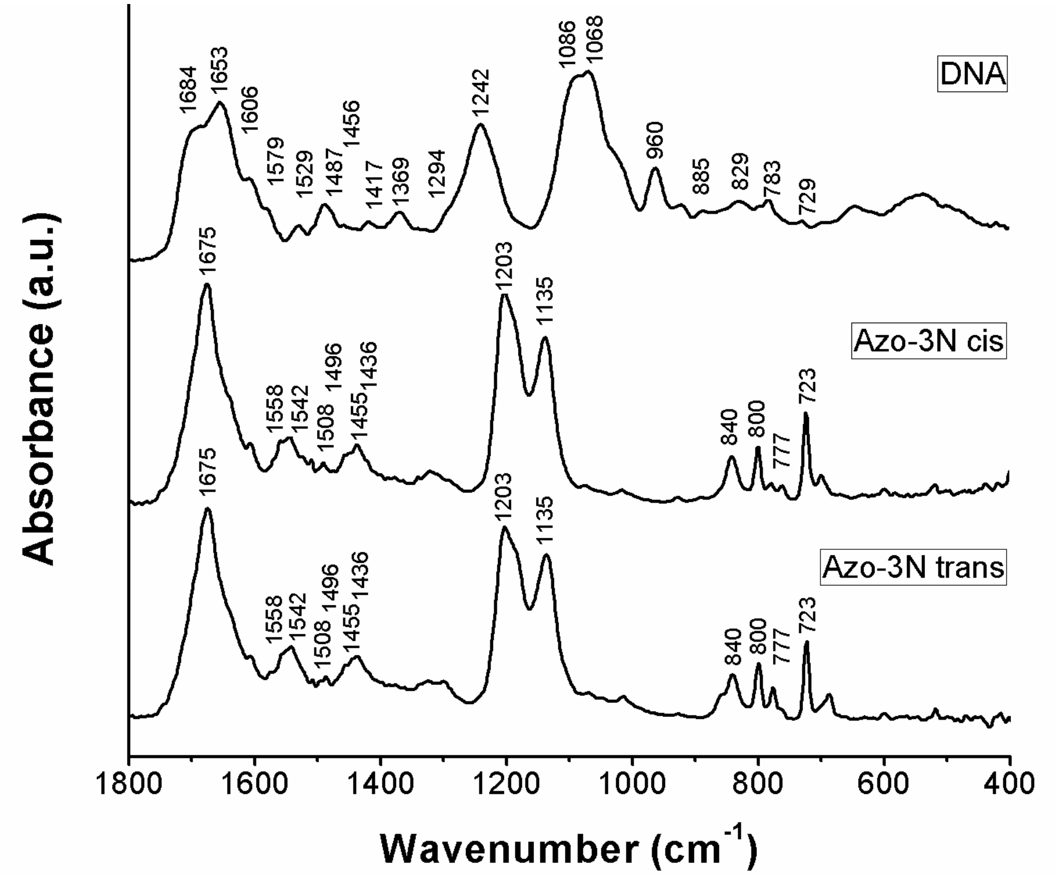

Figure S11. FT-IR spectra of free DNA and Azo-3N in its isomeric forms in the 1800-400 cm^{-1} region.

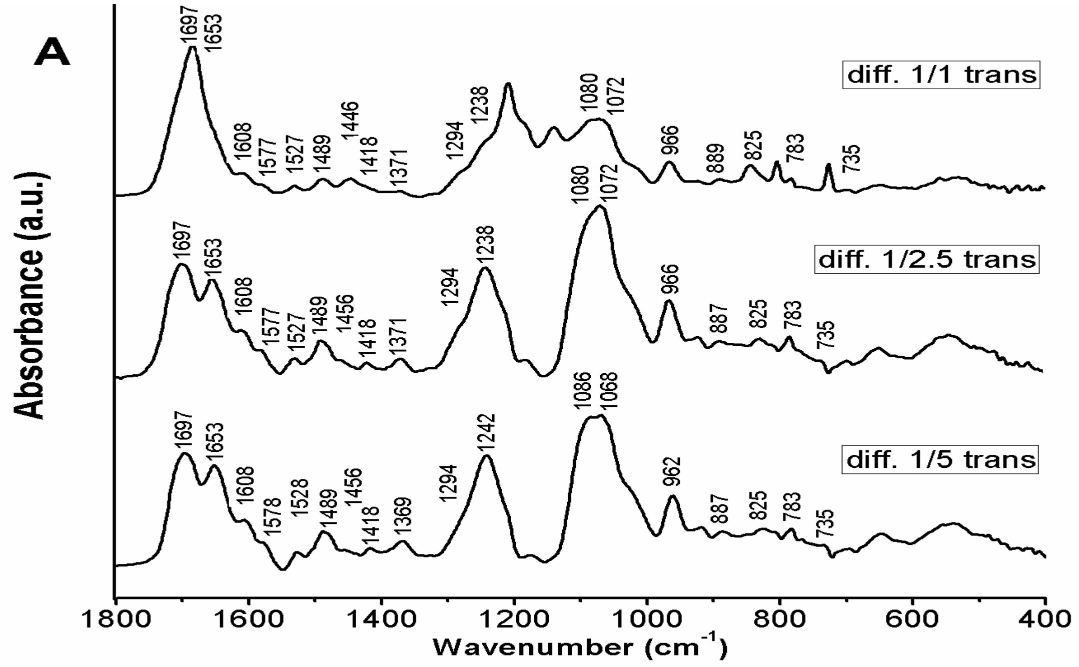

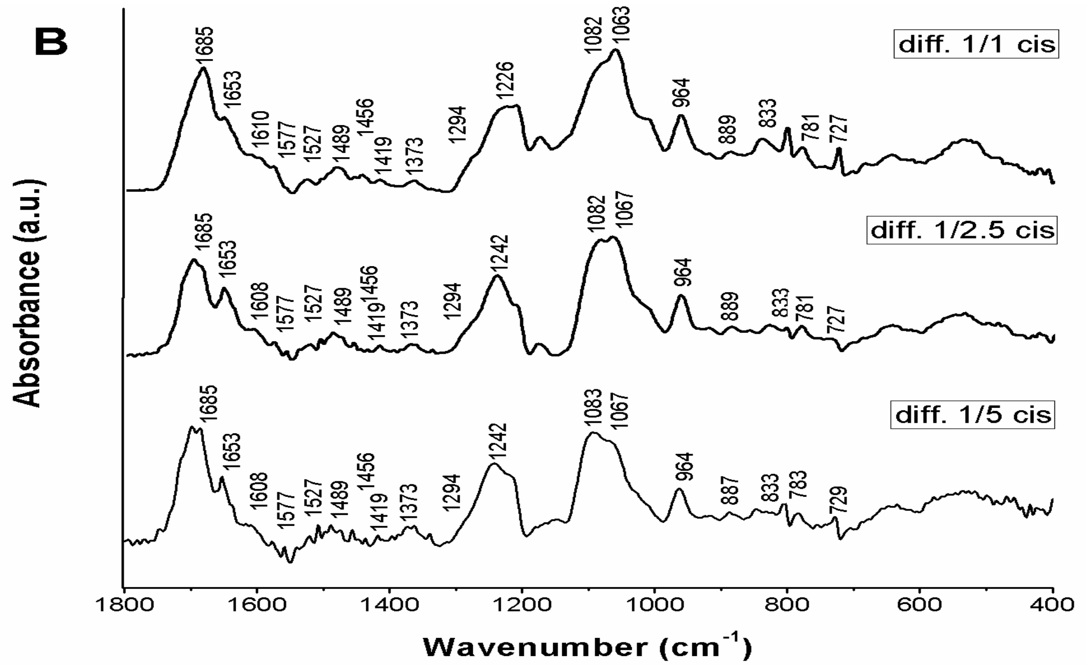

Figure S12. (**A**) Difference FT-IR spectra [(DNA solution + Azo-3N trans) – Azo-3N trans] at different molar ratios in the 1800-400 cm^{-1} region. (**B**) Difference FT-IR spectra [(DNA solution + Azo-3N cis) – Azo-3N cis] at different molar ratios in the 1800-400 cm^{-1} region.

References

[1] Gaussian 09, Revision D.01, Frisch, M. J. *et al.* Gaussian, Inc., Wallingford CT, (2009).

[2] Zhao, Y. & Truhlar, D. G. The M06 suite of density functionals for main group thermochemistry, thermochemical kinetics, noncovalent interactions, excited states, and transition elements: two new functionals and systematic testing of four M06-class functionals and 12 other functionals. *Theor. Chem. Acc.* **120**, 215-241 (2008).

[3] Tomasi, J., Mennucci, B. & Cammi, R. Quantum mechanical continuum solvation models. *Chem. Rev.* **105**, 2999-3094 (2005).

[4] Taillandier, E. & Liquier, J. Infrared spectroscopy of DNA. *Meth. Enzymol.* **211**, 307-335 (1992).

[5] Loprete, D. M. & Hartman, K. A. Conditions for the stability of the B, C, and Z structural forms of poly(dG-dC) in the presence of lithium, potassium, magnesium, calcium, and zinc cations. *Biochemistry* **32**, 4077-4082 (1993).

[6] Neault, J. F. & Tajmir-Riahi, H. A. Structural analysis of DNA-chlorophyll complexes by Fourier transform infrared difference spectroscopy. *Biophys. J.* **76**, 2177-2182 (1999).

[7] Agarwal, S., Jangir, D. K., Singh, P. & Mehrotra, R. Spectroscopic analysis of the interaction of lomustine with calf thymus DNA. *J. Photochem. Photobiol. B* **130**, 281-286 (2014).

[8] Hackl, E. V. *et al.* Study of Ca^{2+}, Mn^{2+} and Cu^{2+} binding to DNA in solution by means of IR spectroscopy. *J. Mol. Struct.* **408**, 229-232 (1997).

[9] Rafique, B., Khalid, A. M., Akhtar, K. & Jabbar, A. Interaction of anticancer drug methotrexate with DNA analyzed by electrochemical and spectroscopic methods. *Biosens Bioelectron.* **44**, 21-26 (2013).

[10] Paul, A. & Bhattacharya, S. Chemistry and biology of DNA-binding small molecules. *Curr. Sci.* **102**, 212-231 (2012).
